# Neoadjuvant chemotherapy in well-differentiated fetal adenocarcinoma: a case report

**DOI:** 10.1186/1756-0500-7-283

**Published:** 2014-05-05

**Authors:** Sihame Lkhoyaali, Saber Boutayeb, Nabil Ismaili, Meriem Aitelhaj, Fahd Ouchen, Abdellatif Benosman, Hassan Errihani

**Affiliations:** 1 Department of Medical Oncology, National Institute of Oncology, Rabat, Morocco; 2 Department of Medical Oncolgy, Marrakech, Morocco; 3 Department of thoracic surgery, Hospital Avicenne, Rabat, Morocco

**Keywords:** Well-differentiated fetal adenocarcinoma, Chemotherapy, Prognosis

## Abstract

**Background:**

Fetal adenocarcinoma of the lung is a rare subtype of pulmonary adenocarcinoma with a relative estimated incidence of 0.5% or fewer of all lung cancers. Because of its extreme rarity, there have been no controlled clinical trials investigating treatment regimens for fetal adenocarcinoma and, as a result, there are no guidelines for management.

**Case presentation:**

We report a case of a well-differentiated fetal adenocarcinoma, which is a variant of pulmonary blastoma, that is a low-grade malignancy and associated with a good prognosis. A 29-year-old Moroccan man presented with a well-differentiated fetal adenocarcinoma staged T3N0M0, who received 3 cycles of neoadjuvant chemotherapy followed by surgery, with no recurrence at 2 years follow-up.

**Conclusion:**

Fetal adenocarcinoma is a rare suptype of adenocarcinoma. Surgical resection is the treatment of choice for resectable disease. The role of chemotherapy in the neoadjuvant setting or adjuvant setting is not well defined.

## Background

Fetal adenocarcinoma (FA) of the lung is a rare subtype of pulmonary adenocarcinoma that exhibits the same tissue architecture and cell characteristics to fetal lung tissue in microscopic examination. It was considered as a variant of blastoma but according to the latest World Health Organization (WHO) classification (2004), it is currently considered as a variant of solid adenocarcinoma with mucin production [1].

FA is a rare tumor, with a relative estimated incidence of 0.5% or fewer of all lung cancers [2-5]. Because of its extreme rarity, there have been no controlled clinical trials investigating treatment regimens for FA and, as a result, there are no guidelines for management [5].

Complete surgical resection is the treatment of choice of FA, similarly to all subtypes of non- small cell lung cancer (NSCLC) [6]. Some case reports suggest that FA is rarely sensitive to chemotherapy (CMT) or radiotherapy [2]. An anecdotal case report shows that chemotherapy with UFT® (tegafur uracil) may be useful in FA [7].

We report a case report of FA managed by neoadjuvant CMT and surgery.

## Case presentation

**Figure 1:**
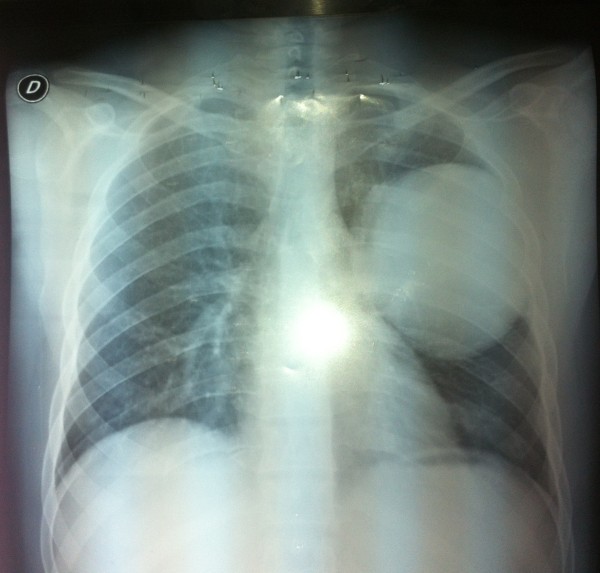
Chest X-ray showing a large left pulmonary opacity.

A 29-year-old Moroccan man without medical history, had a five pack-years smoking history and stopped smoking seven years ago. He presented with a four-month history of chest pain and minimal hemoptysis. The physical exam was normal. A chest X-ray (Figure 1) showed a large left well- rounded pulmonary opacity.

**Figure 2:**
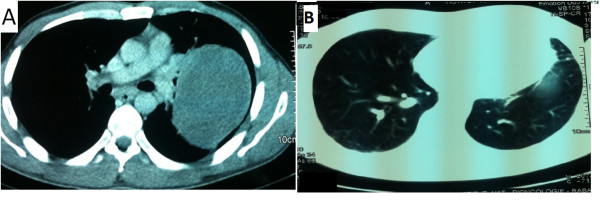
**Chest computed tomography scan. (A)**: lesional process of the left upper lobe before treatment. **(B)**: No signs of recurrence after 2 years.

A chest computed tomography (CT) scan showed a tumoral lesion at the left upper lobe measuring 12.5 × 8 cm. This process was well limited, heterogeneous containing calcifications and areas of necrosis extended into the chest wall without bone lysis or lymph nodes (Figure 2A) staged T3N0.

**Figure 3:**
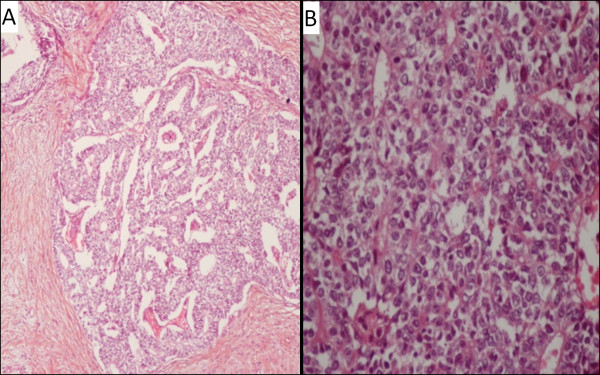
**Microscopic examination of trans-thoracic biopsy showing a numerous glands showing an endometrioid morphology, occasional squamoid morules and relatively clear cytoplasm. (A)**: hematoxylin-eosin staining, original magnification ×10. **(B)**: hematoxylin-eosin staining, original magnification ×40.

Bone scintigraphy, cerebral and abdominal CT scan showed no evidence of distant metastasis. Trans-thoracic biopsy was performed, histopathological study of the biopsy specimen showed a numerous glands showing an endometrioid morphology, occasional squamoid morules and relatively clear cytoplasm, the Ki-67 was 1% (low proliferation index) and based on these histopathological findings, the diagnosis of fetal adenocarcinoma was concluded (Figure 3), then the patient received neoadjuvant CMT based on etoposide at dose of 120 mg/m2 day one to three and cisplatin at 100 mg/m^2^ on day one every three weeks. Evaluation after three cycles showed disease stabilization according to Response Evaluation Criteria In Solid Tumors (RECIST) (as immunohistochemistry was unavailable).

**Figure 4:**
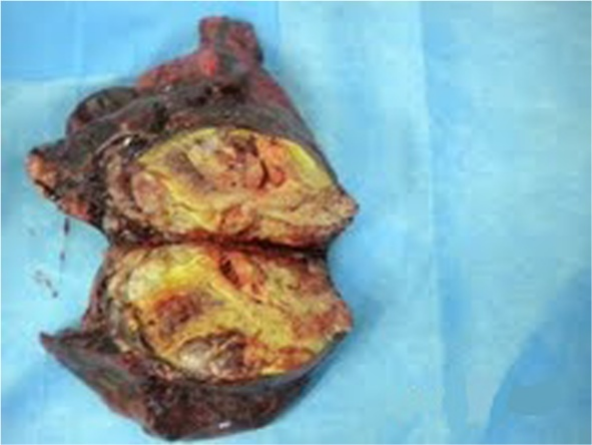
Macroscopic appearance of the tumor.

Thirty days after the last cycle of CMT the patient underwent a left upper lobectomy with lymphadenectomy (Figure 4). The microscopic examination of the specimen found a tumor characterized by two component lesions, the first composed with well-differentiated elements of glandular proliferation rarely isolated or grouped in clusters (polyadenoid structures), which were sometimes separated by undifferentiated beaches of cells with blastomatous differentiation showing moderate cytonuclear atypia and a high mitotic activity. The tumor resection was complete with negative lymph node. The postoperative course was without anomalies.

The patient underwent a chest CT scan assessment every 3 months during the first year and then every 6 months during the second year. At 2 years of follow-up, there is no evidence of disease recurrence (Figure 2B).

## Discussion

In 1982, Kradin *et al.* described fetal adenocarcinoma firstly as a very rare subtype of malignant lung cancer [8]. In pulmonary blastoma, sex distribution was equal and the peak incidence was (35-40 years) of age, 80% of patients are smokers [4]. These are being commonly diagnosed at an early stage without involvement of lymph node.

At the histological level, the element of WDFA characteristically shows glandular elements with tubules composed of glycogen-rich, non ciliated cells that resemble fetal lung, tubuless and squamoid morules may be seen with clear nuclei within lumens. The immature mesenchyme and epithelium mimic the embryonic lung at 10-16 weeks gestation [9,10].

Surgical resection is the standard treatment for patients with pulmonary blastoma [4,5], despite the fact that there have been reports of limited success with adjuvant radiotherapy and chemotherapy [5].

The prognosis of pulmonary blastomas is poor, although prognosis of WDFA is better than biphasic [11], particularly when resection is complete, with a mortality rate of 14% and 52% respectively [4].

Our understanding of fetal adenocarcinoma is derived from case reports and small series. Koss *et al.* reported 28 cases of WDFA, with long follow up of 95 months, and 5-year survival of 81%. Sato *et al.* reported 25 cases; the vast majorities has small size and were N0 at 88% of cases [11,12]. Van Loo *et al.* previously reported 9 cases of WDFA there were beyond T3 stage and underwent postoperative or radiation therapy however the benefit of this treatment was unclear [13].

Our patient received 3 cycles of etoposide – cisplatin protocol. We chose this protocol because of the sensibility of fetal tumors to the combination based on etoposid and cisplatin-like nephroblastoma. Tumor response was assessed by chest CT scan after 3 cycles of CMT, and we showed 12% reduction of the tumor mass corresponding to a stable disease according to RECIST Criteria, this is due to a low proliferation of his tumor (index of proliferation, Ki-67 was 1%).

Thus the role of neoadjuvant CMT in WDFA remains unknown, but we conclude that CMT is ineffective probably because the tumor is not proliferative, however it should be noted that Zaidi [14] has reported a case of WDFA treated with neoadjuvant CMT with mitomycin, ifosfamide and cisplatin for a WDFA staged T4N0M0 in a woman of 27 years, which successfully downstaged the tumor before surgical resection, with good control at 29 months. Also Chanhee *et al.* reported the first case of locally advanced WDFA treated with concurrent chemoradiation therapy with docetaxel with partial response [15]. But we have no idea of the proliferation index, and the cornerstone of treatment is surgery as much as possible.

## Conclusion

Fetal adenocarcinoma is a rare suptype of adenocarcinoma with a better prognosis and is most commonly diagnosed at early stage with no lymph node involvement. Surgical resection is the treatment of choice for resectable disease. The role of CMT in neoadjuvant setting or adjuvant setting is not well defined.

## Consent

Written informed consent was obtained from the patient for publication of this Case report and any accompanying images. A copy of the written consent is available for review by the Editor-in-Chief of this journal.

## Abbreviations

FA: Fetal adenocarcinoma; WDFA: Well-differentiated fetal adenocarcinoma; WHO: World health organization; NSCLC: Non small cell lung cancer; RT: Radiotherapy; CMT: Chemotherapy; CT: Computed tomography; RECIST: Response evaluation criteria in solid tumors.

## Competing interests

The authors declare that they have no competing interests.

## Authors’ contribution

SL was involved in the analysis of the data and the literature research, and she also wrote the manuscript, SB and MA helped with the literature research. NI helped with the patient management and revision of the manuscript. FO and BA contributed to the surgical treatment, HE approved the treatment and analyzed the literature data. All authors read and approved the final manuscript.
